# Hospital Meetings

**Published:** 1904-02-27

**Authors:** 


### HOSPITAL MEETINGS.

### QUEEN CHARLOTTE'S HOSPITAL.

THE annual meeting of the Governors of this hospital was held on Monday last, Mr. F. W. Hunt, vice-president and chairman of the Finance Committee being in the chair. There were also present Mr. R. Martin, Mr. Spyro Mavrojani, Mr. H. J. Royds, Dr. Griffith, and Captain Norcliffe Gilpin. The usual routine of business having been despatched, the chairman announced that under the will of the late Mr. Dresden the hospital received a legacy amounting in the first place to £5,000, to be devoted to the general purposes of the hospital and not to be used for building and furniture. Subsequently when the estate was realised they would be entitled to one-fifth of the residue, amounting probably to £30,000, and another sum would fall in on the death of Mr. Dresden's sister, of which they would also receive one-fifth. The receipt of the £5,000 was conditional on the hospital's acceptance of the £30,000 to be administered by trustees exclusively for the assistance of needy and deserving in-patients on their discharge from the hospital, payments to be made only once a year. A resolution was passed accepting the trusteeship of the £30,000 for the purpose named.

The report for 1903 showed considerable growth in the work of the charity. The total of in-patients was 1,444, being an increase of 173, and by far the largest number ever received in one year. The daily average of patients was 49. Seven patients died, all of whom were admitted with some of the gravest complications of childbirth. There was a very large increase in the out-patients' department also, 1,577 women being delivered in their own homes as against 1,204 in the previous year. Among all these there was only one death. The expenditure amounted to £5,495, which was an increase of £312, due to the greater number of patients and the additional attendance of out-patients. The ordinary income was £4,000, an increase of £229, but still insufficient by £995 to meet the expenditure. £500 had been received for the hospital from King Edward's Hospital Fund and £50 for the Convalescent Home, whilst £476 and £140 had been received from the Sunday and Saturday Funds respectively. The work of the Midwifery Training School had been well maintained, the number of applicants being more numerous than ever. Five months is now the minimum course for midwives. The hospital had been approved by the Central Midwives Board as a training school for midwives, and the committee had been able to arrange for such a course of training as would fully meet all the requirements. The Central Midwives Board had resolved to accept the midwifery certificate of the hospital as a sufficient qualification for a certificate as a midwife. The committee made an earnest appeal for contributions to complete the amount required for an enlargement of the nurses' home. A concert will be held at the Queen's Hall on May 5 on behalf of the hospital, at which Madame Melba has promised to sing.

### ST. MARY'S HOSPITAL FOR SICK CHILDREN, PLAISTOW.

THE annual general meeting of the Governors of this hospital was held on Tuesday last, the Rev. T. Given Wilson being in the chair.

The adoption of the draft report was moved by Councillor Adamson, who commented favourably on the excellent work done by the hospital in the past year, and affirmed his belief that with additional accommodation they would be able to do double the work in the future.

The business of the meeting having been despatched, a vote of thanks was moved to the chairman, who, in replying, said that all the friends and supporters of the hospital ought to make a special effort to raise the sum of money required for the extension. They had now about £4,000, but the total would be £25,000, as it would be folly to build anything but a really good hospital, that was up to date in every way. Such a hospital would be a great boon to the neighbourhood. They already had a splendid site. He further stated that his daughter had raised, by means of a Garment Fund in the parish, the sum of not less than £1'000, which was to be devoted to the endowment of a cot to be called the "Dorcas Cot."

The draft report for the past year showed an increase in the number of admissions to the hospital, which would have been larger but for outbreaks of measles. The number of in-patients was 588, out-patients 3,333, and casualty patients 12,614. The average period of residence of each patient was 17·80 days, while the average cost of each in-patient per week was £1 4s. 8d. During the past year the whole of the cots of the hospital have been quite full on several occasions, and consequently many cases, greatly in need of assistance have had to be sent to other hospitals or treated as out-patients. The income for 1903 exceeded the expenditure by £271. Grants were made by the King's Hospital Fund £600, Hospital Sunday Fund £281, and Hospital Saturday Fund £93.

### THE NATIONAL REFUGES.

#### MEETING AT THE MANSION HOUSE.

A SPECIAL MEETING to advocate the claims of the training ships *Arethusa* and *Chichester* was held at the Mansion House on Monday, the 22nd inst. The Lord Mayor, who has been a member of the Training Ships Committee since 1888, and has long taken a strong personal interest in the work, was in the chair. He was accompanied by the Lady Mayoress, Miss Ritchie, and there were also present on the platform the Bishop of Rochester, Admiral the Hon. E. R. Fremantle, G.C.B., the Rt. Hon. Admiral Sir John Dalrymple

Hay, G.C.B, Sir T. Fowell Buxton, Sir W. Vincent, Sir John Knill, Commander Dawson, Mr. G. H. Morrell, M.P., and others. A guard of honour was formed by 100 boys from the *Arethusa*, who were accompanied by their brass band. There was a large attendance, the Egyptian Hall being well filled.

The Lord Mayor, in opening the proceedings, said the work on these ships was in no sense reformatory, the candidates for training being most carefully selected, and being required to furnish written certificates of good character. During 1903, 101 entered the Royal Navy, while 85 per cent. had joined either that service or the Mercantile Marine. It was absolutely essential that the equipment of men forming the first line of National Defence should be complete, ample, and, he thought, respectable.

The Bishop of Rochester then proposed a resolution to the effect that the work of the training ships, instituted by the seventh Earl of Shaftesbury, deserves hearty and continued support, inasmuch as it stretches out a helping hand to the poor boys in the city, and throughout the United Kingdom, rescues them from the perils of destitution, and trains them to become useful, God-fearing men. Having warmly endorsed the Chairman's remark as to the good character of the boys, the Bishop, in whose diocese the ships are situated, referred to the large number of boys offering themselves for confirmation. The work among them could only be described, he said, as a delightful one, and one that was full of hope. The difficulties of the life for which most of these boys were destined were great, but the training they received in diligence, industry, and self-respect was admirably calculated to render them good citizens, and he could not too highly appraise the value of the religious instruction they received on board, which was very favourably reported upon by the Diocesan Inspector. Everyone must realise the enormous amount of wastage resulting from our present complex civilisation, and there was no greater obligation upon the community than to reduce it by every possible means, one of which was to give the young an opportunity of being trained to take an honourable place in the world. What was wanted was more zeal for righteousness, and a greater degree of personal service. There was room| for three or four more such ships as the *Arethusa* and *Chichester*. Thousands of boys were waiting to be given a start in life, and the community must not sit down with folded hands because here and there a few hundreds were being helped. He heartily commended the work of the ships to the support of the public.

Captain G. King Hall having seconded, and spoken in very favourable terms of the ships, which he had recently inspected on behalf of the Admiralty, the resolution was carried.

Admiral Fremantle and the Rev. W. Bryant Salmon having spoken in support of the Society, which has already sent 7,000 poor boys into the Royal Navy and Merchant Service, Sir John Dalrymple Hay proposed that the cordial thanks of the meeting be tendered to the Lord Mayor for his warm support of the National Refuges' Training Ships, and for presiding. Allusion having been made by a previous speaker to a lecture delivered by himself at the United Service Institution recently, Sir John Hay said there were 49,800 foreign seamen in the British Navy, occupying places which ought to be filled by British subjects, trained on such ships as these; and he thought much of the present day lack of employment would be obviated if boys were trained in larger numbers for the service. As things were, foreigners represented one fifth of the persons employed, and the proportion was increasing. The 10,000 boys constantly under training should be supplemented by an equal number for the Mercantile Marine.

Mr. W. Klein announced that the annual fête-day would take place in June, when visitors would be welcome and the work of the Society could be inspected. On behalf of the Committee, he warmly seconded the vote of thanks.

The Lord Mayor, in replying, deprecated the substitution of technical schools for the old apprenticeship system, under which he thought boys learnt their trade much more thoroughly; he agreed with Sir John Hay that an extension of training such as that given on the *Arethusa* and *Chichester* was one method of dealing with the lack of employment. He was sure the boys, many of whom were present, were deeply grateful for the training which enabled them, when opportunity arose, to go forth and do good service for their country as citizens of this great empire.

At the close of the meeting a collection was made in aid of the ships, which are worked entirely on voluntary principles for poor boys of good character. Since 1866, 7,458 boys have been prepared for a seafaring life, of whom 5,364 have entered the merchant service, 1,060 have joined the Royal Navy, while the remainder have joined the Army, Royal Marines, or other service.

